# Chemical Stability of the Fertilizer Chelates Fe-EDDHA and Fe-EDDHSA over Time

**DOI:** 10.3390/molecules26071933

**Published:** 2021-03-30

**Authors:** Ewelina Klem-Marciniak, Marta Huculak-Mączka, Kinga Marecka, Krystyna Hoffmann, Józef Hoffmann

**Affiliations:** 1 Department of Engineering and Technology of Chemical Processes, Faculty of Chemistry, Wroclaw University of Science and Technology, Wybrzeże Wyspiańskiego 27, 50-370 Wrocław, Poland; marta.huculak@pwr.edu.pl (M.H.-M.); 234168@student.pwr.edu.pl (K.M.); jozef.hoffmann@pwr.edu.pl (J.H.); 2 Department of Micro, Nano and Bioprocess Engineering, Faculty of Chemistry, Wroclaw University of Science and Technology, Wybrzeże Wyspiańskiego 27, 50-370 Wrocław, Poland; krystyna.hofmann@pwr.edu.pl

**Keywords:** Fe-EDDHA, Fe-EDDHSA, micronutrient fertilizer, stability, degree of ions complexation

## Abstract

In application conditions, the influence of environmental parameters on used fertilizer chelates and their distribution over time is important. For this purpose, the changes in the content of micronutrient ions and Fe-EDDHA and Fe-EDDHSA chelates in an aqueous medium at different pH values were studied. In the assumed time, changes in the ions content were analyzed using the voltammetry method at pH 3, 5 and 7. The content of isomers and chelate forms was analyzed by ion pair chromatography at pH 3, 5 and 7. These studies allowed us to determine the effect of pH on the stability of iron chelates over time.

## 1. Introduction

The increase in humanity has resulted in an increase in the demand for food, and thus the need to intensify agriculture. Due to the quality of plants and their yields, it is necessary to supply plants with macro- and microelements during their various stages of vegetation. One of the most important factors determining nutrient uptake from soil and affecting plant growth and yield is pH. Due to the increase in the acreage of arable land, crops are often extended to limestone and alkaline soils. Under high pH conditions, the micronutrients appear in a form that is not absorbed by plants. Micronutrients are applied in fertilization processes in the form of inorganic salts, as an addition to macronutrient fertilizers, fertilizing glazes and chelates [1,2,3,4].

The most effective way to supplement the deficiency of micronutrients is the use of micronutrient chelates. The effectiveness of their operation is related to the structure of the molecule. Chelates are resistant to the action of microorganisms, and they stabilize the microelement in a wide pH range. Their use, even in the absence of micronutrient deficiencies, increases the yield. In-soil application of micronutrients in the form of ions is often associated with the risk of these metals becoming non-available by plants, which results in the use of several times higher doses for soil fertilization than for foliar fertilization. The chelate structure prevents the microelement ions from reacting with the components of the soil solution, protects it against regression, and facilitates the uptake of nutrients by the leaves. Chelates are very soluble in water, but only slightly dissociate. The gradual release of micronutrients increases their absorption by plants and prevents their excessive uptake [5,6,7]. Regulation (EC) No 2019/1009 of the European Parliament and of the Council of 5 June 2019 on fertilizers legalized fertilising chelators of synthetic origin and complexing substances of natural origin (lignosulfonic acid salts). Synthetic substances with chelating properties belong to the group of aminopolycarboxylic compounds. Aminopolycarboxylic acid (APCA) compounds are characterized by high durability constants and effective supply of nutrients to plants [8]. They form complexes with micronutrient cations in a molar ratio of 1:1. The most commonly used chelating agent is EDTA (ethylenediaminetetraacetic acid or its salt). Despite the good properties of chelating metal ions and their use in many branches of industry, chelators belonging to the group of aminopolycarboxylic compounds are characterized by a low degree of biodegradation. Their high concentration is found in European surface waters. They pose a risk of binding heavy metal ions and introducing them into the food chain [8,9,10,11,12,13].

The requirements of the European Directive define the content of water-soluble, available micronutrient ions. Micronutrient fertilizers should contain a minimum of 80% of the total water-soluble amount of metal ions chelated by a specific chelating agent; 50% of the water-soluble trace element should be chelated by a chelating agent approved by the European Parliament and the Council. The official analytical methods included in the Directive have also been standardized to determine the content of individual chelates in commercial products. For such analyzes, high-performance liquid chromatography (HPLC) or ion chromatography are used, which are coupled to an ultraviolet–visible (UV–Vis) detector. Ion pair chromatography (ethylenediamine-*N*,*N*′-di[(*ortho*-hydroxyphenyl)acetic acid] *o*,*o*-EDDHA, ethylenediamine-*N*,*N*′-di[(*ortho*-hydroxy-methylphenyl)acetic acid] *o*,*o*-EDDHMA, ethylenediamine-*N*,*N*′-di[(2-hydroxy-5-sulfophenyl)acetic acid] and its condensation products EDDHSA, *N*,*N*′-di(2- hydroxybenzyl)ethylenediamine-*N*,*N*′-diacetic acid HBED), ion chromatography (ethylenediaminetetraacetic acid EDTA, ethylenetriaminepentaacetic acid DTPA, 2-hydroxyethylethylenediaminetriacetic acid HEEDTA) and reverse phase chromatography (ethylenediamine- *N*-[(*ortho*-hydroxyphenyl)acetic acid]- *N*′-[(*para*-hydroxyphenyl)acetic acid] *o*,*p*-EDDHA) are used. The lack of a unique, official method for the simultaneous determination of different chelating agents is due to the low selectivity of the UV–Vis detector. Good chromatographic separation is necessary, especially for structurally related compounds. The formal and legal approval of official analysis methods increased the content of chelated microelements in fertilizers, and thus their quality [8,14,15,16].

**Figure 1:**
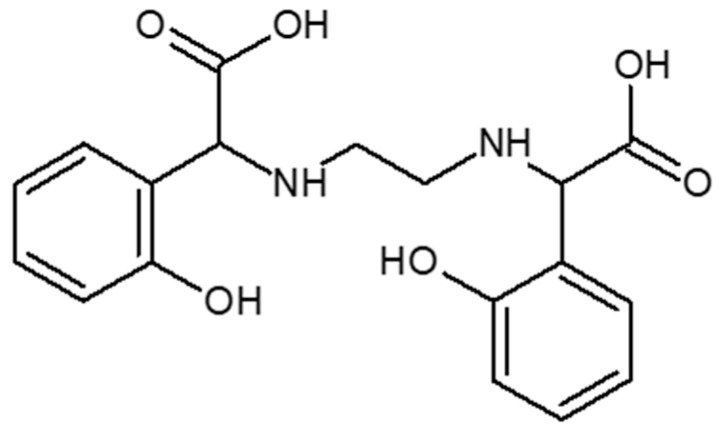
Structure of ethylenediamine-*N*,*N*′-di[(*ortho*-hydroxyphenyl) acetic] ([*o*,*o*] EDDHA).

**Figure 2:**
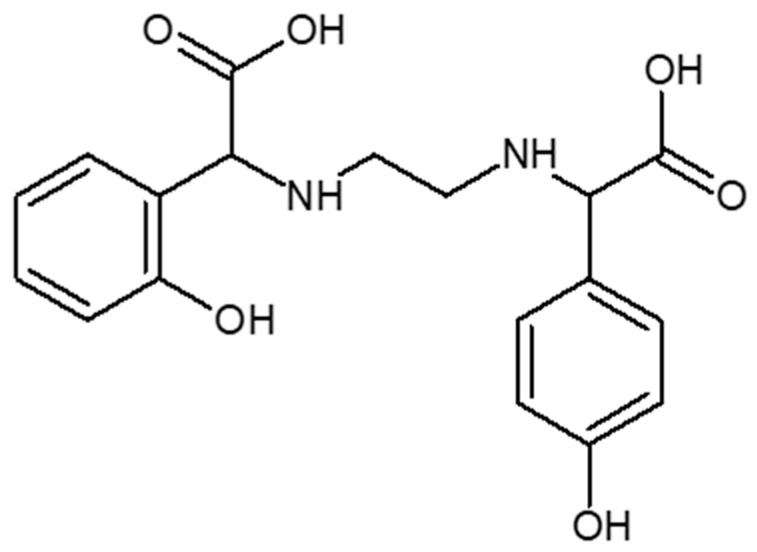
Structure of ethylenediamine-*N*-[(*ortho*-hydroxyphenyl) acetic acid]-*N*′-[(*para*-hydroxyphenyl) acetic acid ([*o*,*p*] EDDHA).

**Figure 3:**
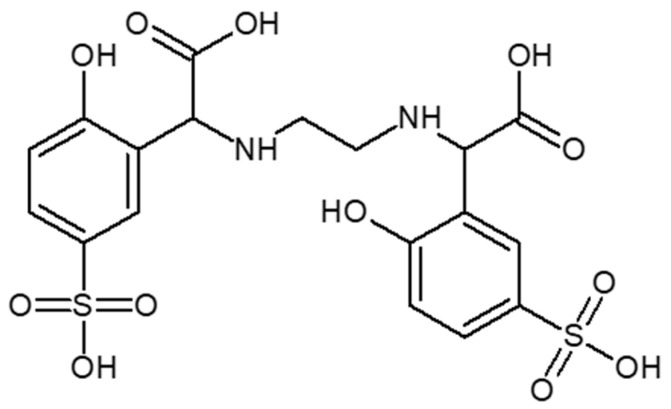
Structure of ethylenediamine-*N*,*N*′-di[(2-hydroxy-5-sulfophenyl) acetic acid] (EDDHSA).

The problem in alkaline soils is the deficiency of bioavailable iron compounds, which in such an environment creates water-insoluble compounds that are not absorbed by the plant. The lack of this trace element causes chlorosis. Ethylenediamine-*N*,*N*′-di[(*ortho*-hydroxyphenyl) acetic] ((*o*,*o*) EDDHA or EHPG)) and its analogues are considered the most effective chelators used to replenish iron deficiency in neutral and alkaline soils and combating chlorosis. Ethylenediamine-*N*,*N*′-di[(*ortho*-hydroxyphenyl) acetic] ([*o*,*o*] EDDHA) (Figure 1) and ethylenediamine-*N*-[(*ortho*-hydroxyphenyl) acetic acid]-*N*′-[(*para*-hydroxyphenyl) acetic acid ([*o*,*p*] EDDHA) (Figure 2) or salts of these acids and ethylenediamine-*N*,*N*′-di[(2-hydroxy-5-sulfophenyl) acetic acid] (Figure 3) and condensation products or their salts (EDDHSA) complex cations of micronutrients over a wide pH range. They effectively provide micronutrients to plants, enabling them to supplement their deficiency, also in soils with high pH [17,18,19].

EDDHSA and its condensation products are used to correct iron deficiencies and combat the effects of chlorosis. Fe-EDDHSA shows a similar effectiveness as its derivatives. The solubility of Fe-EDDHSA is about 3.4 times greater than that of Fe-EDDHA. The presence of sulfo groups makes the phenolic groups more acidic, which results in an increase in the affinity of iron for this chelator. The amount of chelated iron in Fe-EDDHSA and its solubility make it possible to use it at a dose of 1.4–1.7 lower than that of Fe-EDDHA, showing similar effectiveness and stabilizing microelement at pH 4–9 [16,17,18,19].

EDDHA and EDDHSA chelators can be used to supplement iron deficiency at all pH values. The EDDHA ligand comes in three forms (*ortho*, *ortho*), (*ortho*, *para*), (*para*, *para*). The (*o*,*o*) EDDHA isomer has the best complexing properties, has six binding sites, and forms stable chelates. The isomer (*ortho*, *para*) has five bonding sites. It is characterized by lower stability, but also faster delivery of micronutrient ions to the plant. The presence of both isomers in fertilizers causes both a gradual, long-lasting release of metal ions ((*o*,*o*) isomer) and quick action ((*o*,*p*) isomer). The steric hindrance causes (*p*,*p*) EDDHA to show no complexing properties. In the case of EDDHSA, no constitutional isomers are formed [20,21,22,23,24,25,26].

On an industrial scale, a one-step Mannich reaction is used to obtain EDDHA and EDDHSA iron chelates. Chelates made by this method often contain a large amount of by-products, unreacted substrates, and positional isomers [27,28].

In the application conditions, the influence of environmental parameters on the preparations used and their distribution over time is important. To the best of our knowledge, so far, no experiments have been conducted to determine the change in the content of Fe-EDDHA isomers and Fe-EDDHSA forms in an aqueous solution depending on pH over time. For this purpose, changes in the content of micronutrient ions and Fe-EDDHA and Fe-EDDHSA chelates at different pH values in an aqueous environment were investigated. At the assumed time, changes in the ion content were analyzed using the voltammetry method at pH 3, 5, 7, 9, 11 and 13. The content of chelates was analyzed by ion pair chromatography at pH 3, 5 and 7. The studies allowed the effect of pH on the stability of iron chelates to be determined [29,30,31]. The obtained results will allow us to determine whether these chelates can be produced and stored in the form of aqueous solutions. This will reduce production costs and facilitate the application.

## 2. Results and Discussion

Fe-EDDHA and Fe-EDDHSA chelates obtained as a result of the Mannich reaction under laboratory conditions were used for the experiments aimed at measuring the content of iron(III) ions and chelates over time. The content of Fe^3+^, Fe-EDDHA, and Fe-EDDHSA was analyzed over time in the aquatic environment. Table 1 shows the initial ion contents in all tested systems.

At pH 7, 96.70% of the iron(III) ions are bound by the EDDHA chelator in the aquatic environment. Under such conditions, the cations of this metal do not undergo any other chemical reactions. In other cases, it is impossible to link their content directly to the degree of complexation. At acidic pH, there is an overlap of the cation peak with that of H_3_O^+^. Iron(III) hydroxide is precipitated in an alkaline environment.

The obtained results of the degree of complexing of iron(III) ions (pH 7) confirm the information contained in the work by Felipe Sun et al. on the complexing ability of EDDHSA. In their work, they presented protonation constants and stability constants for various metal ions. On their basis, they determined that the EDDHSA chelator has a greater ability to chelate iron(III) ions than EDDHA. However, these differences are minor [20].

At a pH of 7, the required degree of ion complexation of more than 80% was achieved. Which, according to Regulation (EC) No 2019/1009 of the European Parliament and of the Council of 5 June 2019 on fertilizers, allows for the preparation of Fe-EDDHA and Fe-EDDHSA chelates using this method. Borowiec et al., conducting research on the degree of complexation of zinc ions by aminopolycarboxylic compounds at various pH values, also obtained a result above the required 80%. The degree of iron (III) ion complexation by EDDHA and EDDHSA compounds containing an aromatic ring in their structure is higher [31].

**Figure 4:**
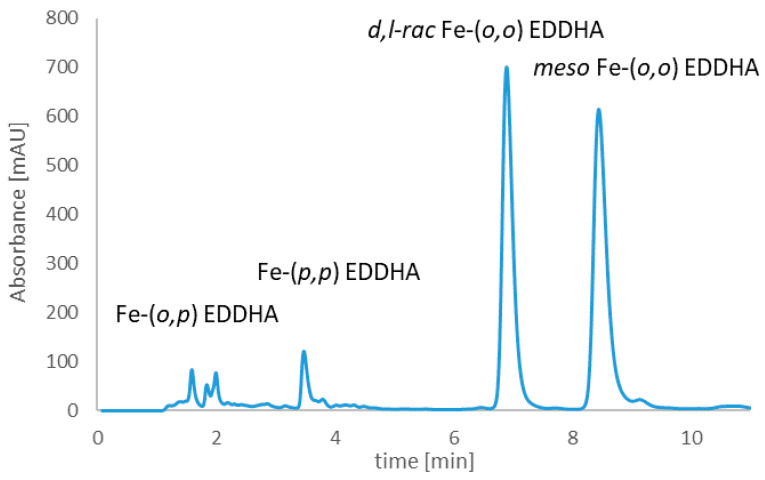
Iron(III) ethylenediamine-*N*,*N*′-bis(hydroxyphenylacetic) acid chelate chromatogram, which was used to study the changes in the content of chelate and iron(III) ions over time.

**Figure 5:**
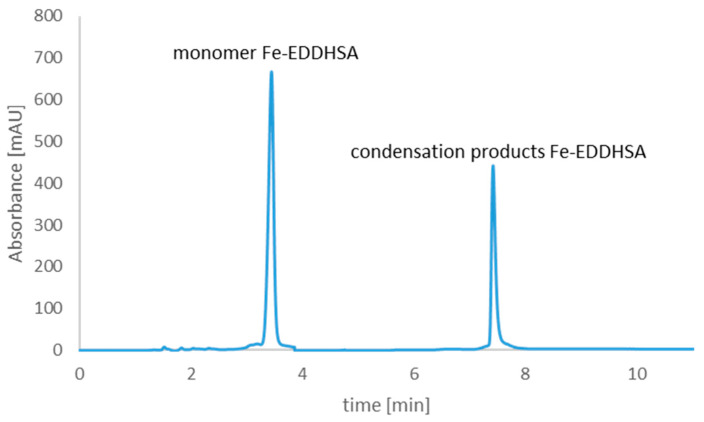
Iron(III) ethylenediamine-*N*,*N*′-di[(2-hydroxy-5-sulfophenyl)acetic acid] chelate chromatogram, which was used to study the changes in the content of chelate and iron (III) ions over time.

Figure 4 and Figure 5 shows the chromatograms of Fe-EDDHA and Fe-EDDHSA chelates, which were used to study the changes in the content of iron ions and chelates over time. The use of such a complex made it possible to determine the stability of individual forms over time at different pH values.

On the basis of the chromatograms, the contents of individual Fe-EDDHA isomers and Fe-EDDHSA forms were calculated. Table 2 shows the initial contents of all Fe-EDDHA isomers in the aqueous solution at pH 3, 5, and 7. Table 3 shows the initial contents of the monomer and Fe-EDDHSA condensation products in the aqueous solution at pH 3, 5 and 7.

The total initial concentration of Fe-EDDHA and the content of individual isomers depend on the conditions of the experiment. The lowest concentration was observed at pH 3. At this pH, Fe-EDDHA is the least stable. The highest total chelate concentration was observed at pH 7. These conditions favor the formation and stability of Fe-EDDHA.

The initial concentrations of these isomers increased with increasing pH. The increase in pH favors the formation of the isomer (*ortho, ortho*). An increase in pH promotes the formation of the isomer (*ortho*, *ortho*). This isomer is most stable at pH 7. The amount of Fe-(*o*,*p*) EDDHA decreased with increasing basicity. The (*o*,*p*) isomer is not less stable than the (*ortho*, *ortho*) isomer in a neutral aqueous environment. The content of the Fe*-(p*,*p*) EDDHA isomer differs depending on the pH value.

The total initial chelate concentration, monomer content, and condensation products will vary depending on the conditions under which the experiment was conducted.

In the aqueous solution, the initial total Fe-EDDHSA concentration was highest at pH 5. In a slightly acidic environment, the metal-ligand reaction shifts towards the formation of chelate. However, slight changes in the content of monomer and condensation products depending on the pH are observed. The ratio of the contents of both forms is close to 1:1.

### 2.1. Iron(III) Ions Content in Analyzed Chelate Samples

During the period of 21 days, changes in the content of iron(III) ions in the aqueous medium at pH 3, 5, 7, 9, 11 and 13 were investigated using the differential pulse voltammetry method. The applied pH range allows the change in iron(III) ion content to be determined, due to the chemical properties of this metal ions, it is not possible to determine changes in the degree of complexation under these conditions.

**Figure 6:**
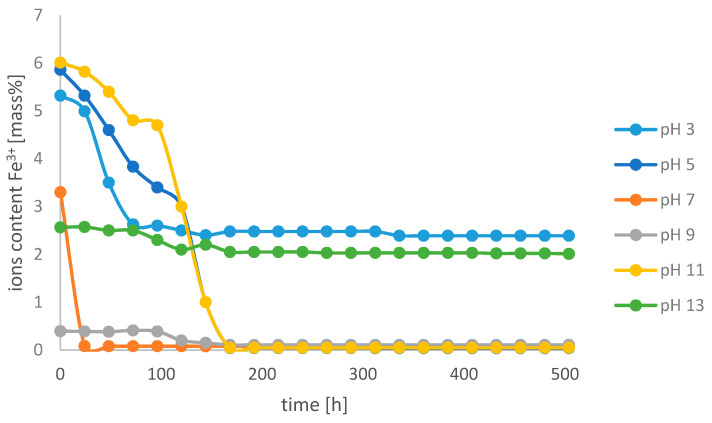
Change in the content of Fe^3+^ ions over time at different pH values in the pH electrolyte environment in the iron(III) ethylenediamine-*N*,*N*′-bis(hydroxyphenylacetic) acid solution.

Figure 6 shows the changes in the content of Fe^3+^ ions in the base electrolyte environment in the Fe-EDDHA chelate sample at different pH values.

The lowest content of iron(III) ions in the aquatic environment was observed at pH 7. On the third day of the experiment, the content of free ions was 0.07%. In such an environment, the unbound iron ions only react with the chelator and do not undergo other reactions. The highest content was observed at pH 3 and 13. The greatest decrease in the content of ions was observed at pH 11, which is caused by the precipitation of iron(III) hydroxide. Within 168 h of the experiment, the content of free iron ions stabilized and did not change significantly until the end of the experiment. The results obtained indicate that the composition of the solution undergoes slight changes over time.

**Figure 7:**
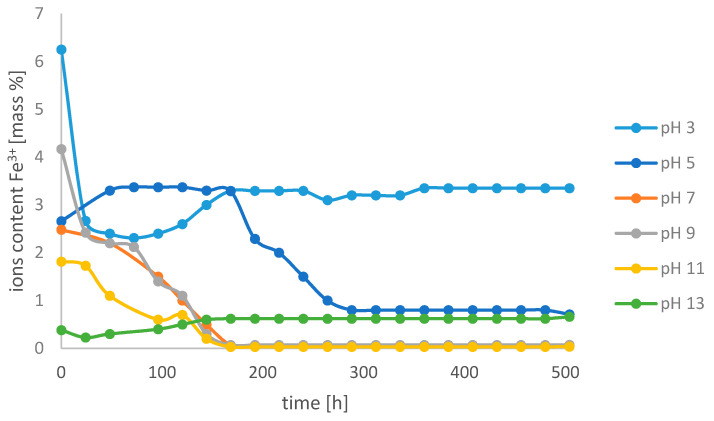
Change in the content of Fe^3+^ ions over time at different pH values in the pH electrolyte environment in the iron(III) ethylenediamine-*N*,*N*′-di[(2-hydroxy-5-sulfophenyl)acetic acid] solution.

Figure 7 shows the changes in the content of Fe^3+^ ions in the base electrolyte environment in the Fe-EDDHSA chelate sample at different pH values.

The highest concentration of free ions was observed at pH 3, and successively at pH 5 and 13. Their content in the case of *t* = 500 h was 3.35%, 0.71%, and 0.66%, respectively. At the basic pH, iron(III) hydroxide is precipitated, therefore a decrease in the content of micronutrient ions is observed. With the remaining pH values and the stabilization of the cation content, the Fe^3+^ concentration was at a similar level, equal to 0.07%. After 168 h of the experiment, the content of free ions at all pH values did not change. The content of free iron ions is small and does not change with time. This allows the choice not to change the word composition of Fe-EDDHSA over time.

### 2.2. Content of Fe-EDDHA (Fe-Ethylenediamine-N,N′-di[(Ortho-Hydroxyphenyl) Acetic) Isomers and Fe-EDDHSA (Fe-Ethylenediamine-N,N′-di[(2-Hydroxy-5-Sulfophenyl) Acetic Acid]) Forms in the Analyzed Samples

In the studies of Fe-EDDHA content in the water environment, changes in the retention time of individual isomers of this chelate with time were observed. The tests were performed for a period of 30 days, in subsequent experiments the retention time of Fe-(*o*,*p*) EDDHA was extended, and the remaining isomers were eluted faster from the column. The retention times varied a little depending on the pH of the system.

**Figure 8:**
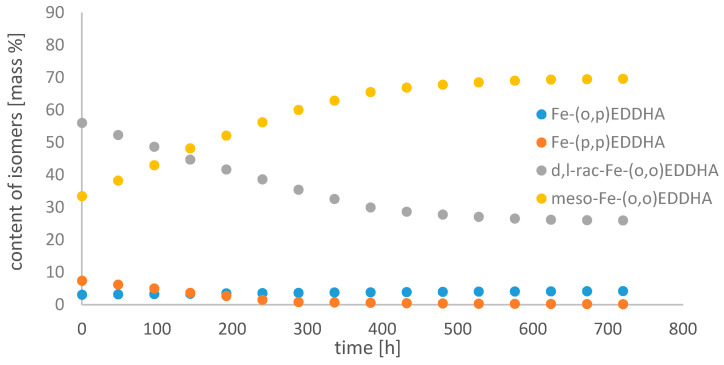
Change in the content of iron(III) ethylenediamine-*N*,*N*′-bis(hydroxyphenylacetic) acid isomers with time in an aqueous solution at pH 3.

**Figure 9:**
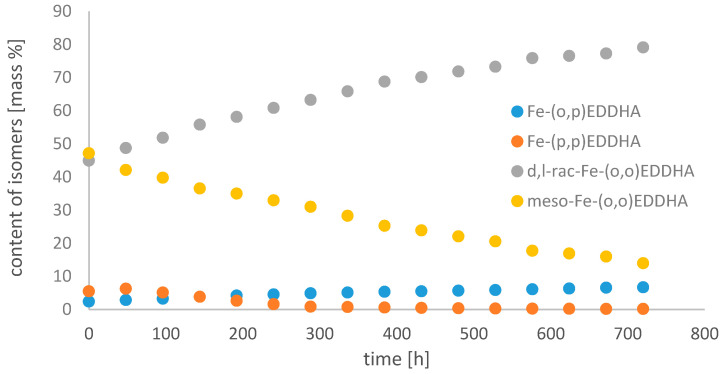
Change in the content of iron(III) ethylenediamine-*N*,*N*′-bis(hydroxyphenylacetic) acid isomers with time in an aqueous solution at pH 5.

**Figure 10:**
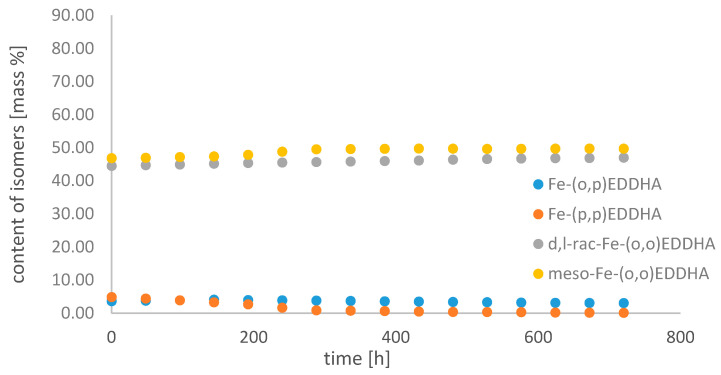
Change in the content of iron(III) ethylenediamine-*N*,*N*′-bis(hydroxyphenylacetic) acid isomers with time in an aqueous solution at pH 7.

Figure 8, Figure 9 and Figure 10 show changes in the content of all Fe-EDDHA isomers during 30 days at pH 3, 5 and 7 in an aqueous solution, calculated on the basis of standard curves.

During the chromatographic determinations, changes in the content of individual isomers depending on the pH were observed. The total chelate concentration increased over time at pH 3. This was probably due to the low solubility of the solution pH used. A slight increase was also observed at pH 7. At pH 5, the total concentration of the complex decreased.

With time, the concentration of the Fe-(*o*,*p*) EDDHA isomer increased slightly, and Fe-(*p*,*p*) EDDHA decreased. The content of the isomer (*para*, *para*) under the conditions of the assay decreased to 0.19% at pH 3, to 0.18% at pH 5, and to 0.15% at pH 7. At pH 3, the concentration of *d,l*-rac-Fe-(*o*,*o*) EDDHA was decreasing. The content of this isomer in the tested conditions decreased from 56.04% to 25.99% during the experiment. At higher pH values, the concentration of this isomer increased at pH 5 from 44.92% to 79.11%, and at pH 7 from 44.55% to 47.02%. The concentration of meso-Fe-(*o*,*o*) EDDHA increased over time at pH 3 and 7. The concentration of this isomer decreased significantly over time at pH 5. Its content decreased from 47.15% to 13.99%.

The results obtained indicate that the composition of the aqueous Fe-EDDHA solution changes little over time. Due to the stability of the isomers, the most advantageous factor is that it is neutral. The obtained results confirm that the (*para*, *para*) isomer is characterized by the lowest stability. Due to the low concentration of this isomer and its distribution over time, it cannot effectively replenish the deficiency of micronutrients in plant tissues [20,23]. The effectiveness of chlorosis treatment is determined by the presence of isomers (*ortho*, *ortho*), both *rac-* and *meso-*. The (*o*,*p*) isomer is present in a small concentration and its content decreases over time, which means that it does not have a large impact on the effectiveness of the chelate. Schenkeveld et al. observed a similar relationship. In their work, they investigated the concentration of iron (III) ions and the content of Fe-EDDHA chelate and its isomers after contact with soil. The results obtained show that to increase the efficiency of iron deficiency supplementation, the EDDHA synthesis should be modified in such a way as to obtain the highest isomer content (*ortho*, *ortho*) [23].

In Fe-EDDHSA stability studies, changes in the elution time of monomer and chelate condensation products from the column were observed. As the pH value increased in the chromatograms, a shorter retention time for monomer and condensate was observed. During the experiment, the retention time of both forms was shortened.

**Figure 11:**
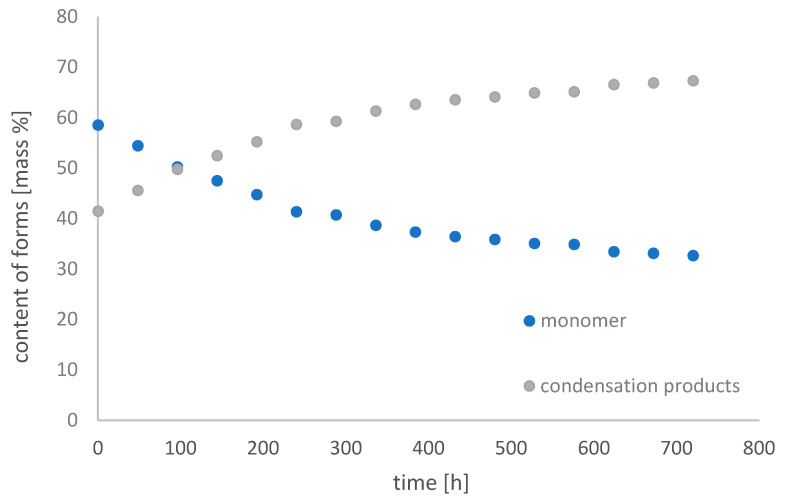
Change in the content of iron(III) ethylenediamine-*N*,*N*′-di[(2-hydroxy-5-sulfophenyl)acetic acid] forms with time in an aqueous solution at pH 3.

**Figure 12:**
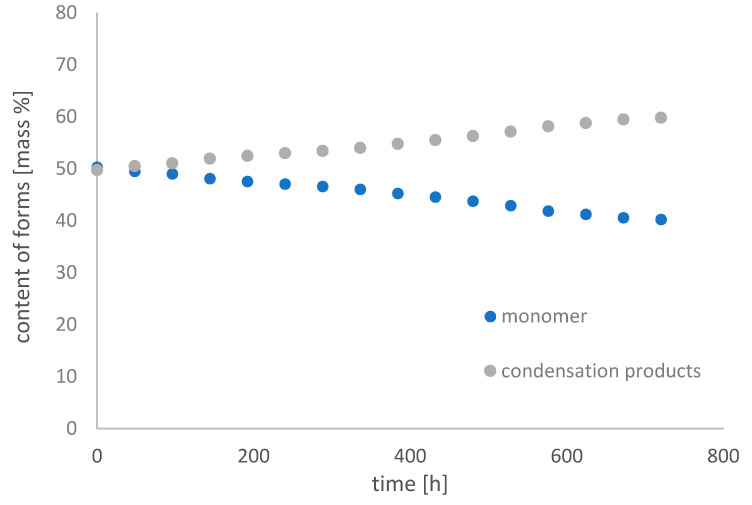
Change in the content of iron(III) ethylenediamine-*N*,*N*′-di[(2-hydroxy-5-sulfophenyl)acetic acid] forms with time in an aqueous solution at pH 5.

**Figure 13:**
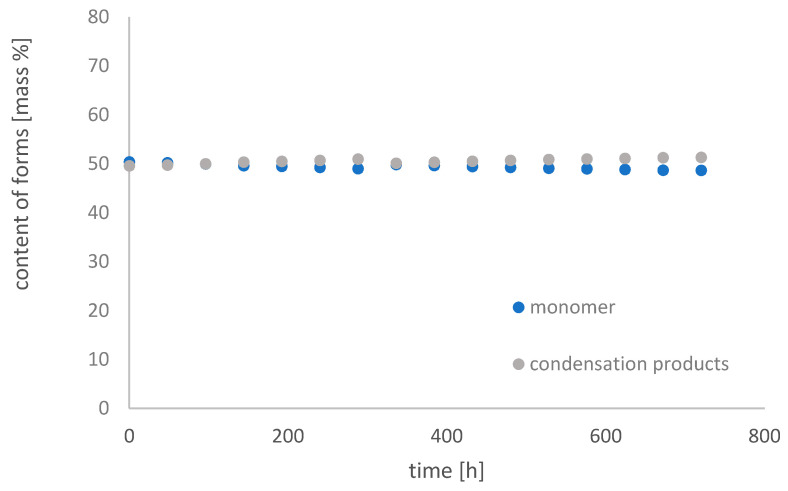
Change in the content of iron(III) ethylenediamine-*N*,*N*′-di[(2-hydroxy-5-sulfophenyl)acetic acid] forms with time in an aqueous solution at pH 7.

Figure 11, Figure 12 and Figure 13 show changes in the content of Fe-EDDHSA monomer and condensation products over 30 days at pH 3, 5, and 7 in an aqueous solution.

The monomer content decreased over time. It underwent condensation reactions in the system used, as a result of which the content of condensation products increases. Such reactions have only been observed in an acidic environment. At pH 3, the monomer content decreased from 58.53% to 32.67%, at pH 5 from 50.28% to 40.21%, and at pH 7 the changes were slight from 50.40% to 48.68%. The greatest changes were observed for 400 h of the experiment. After this time, the contents of both forms did not change significantly. As the pH increases, the changes in the content of both forms are smaller and smaller. A constant content of both forms for a period of 30 days was observed at pH 7. The higher the pH, the total concentration of Fe-EDDHSA changed less and less over time. There was a slight increase in the total chelate concentration over time, possibly related to its solubility.

Both chelate forms, monomer and condensation products, chelating products and their content have no effect on the effectiveness of such micronutrient chelates. The results obtained showed that this chelate was repaired by changes in the neutral test in an aqueous solution during the experiment.

Fe-EDDHA and Fe-EDDHSA chelates in an aqueous medium are the most stable over time at pH 7. Their concentration and the content of EDDHA isomers as well as the content of monomer and EDDHSA condensation products are slightly changed over time. The chelation reaction of iron ions is fast and no significant changes in the content of unbound ions in such a system are observed.

## 3. Materials and Methods

**Figure 14:**
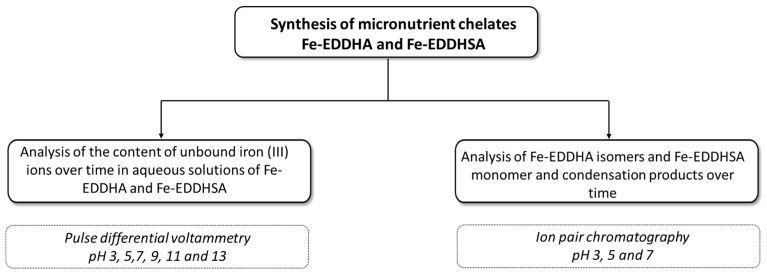
Scheme of analyses performed to determine the chemical stability of Fe-EDDHA and Fe-EDDHSA microelement chelates.

In order to determine the chemical stability of Fe-EDDHA and Fe-EDDHSA micronutrient chelates, the synthesis was carried out under laboratory conditions. Chelates were obtained as a result of a one-step synthesis using the Mannich reaction. Subsequently, at different pH values, the content of free iron (III) ions and Fe-EDDHA isomers and Fe-EDDHSA monomer and condensate products were analyzed. In order to determine the content of iron(III) ions, analyses were performed using the differential pulse voltammetry method. The contents of isomers and forms of both chelates were determined on the basis of analyses performed by ion pair chromatography. The scheme of the performed tests is presented in Figure 14.

### 3.1. Materials

The materials used to perform the synthesis of Fe-EDDHA were: phenol (CAS number 108-95-2) purchased by POCH, Poland, ethylenediamine (CAS number 107-15-3) purchased by purchased by, 50 mass% sodium hydroxide (CAS number 1310-73-2) purchased by POCH and 50 mass% aqueous solution monohydrate glyoxylic acid (CAS number 298-12-4) purchased by MERC MILIPORE, Poland. The solvents used for the separation process were toluene (CAS number 108-88-3, purchased from POCH Poland) and deionized water. The chelation process was performed using iron nitrate nonahydrate (CAS number 10421-48-4, purchased by Sigma-Aldrich, Poland).

The materials used to perform the synthesis of Fe-EDDHSA were: 65 mass% aqueous solution *para*-hydroxybenzenesulfonic acid (CAS number 98-67-9) purchased from Sigma Aldrich Poland, ethylenediamine (CAS number 107-15-3) purchased from purchased by, 50 mass% sodium hydroxide (CAS number 1310-73-2) purchased from POCH, Poland, and 50 mass% aqueous solution monohydrate glyoxylic acid (CAS number 298-12-4) purchased by MERC MILIPORE, Poland. The chelation process was performed using iron nitrate nonahydrate (CAS number 10421-48-4, purchased from Sigma-Aldrich Poland).

In ion pair chromatography, 40 mass% tetrabutylammonium hydroxide aqueous solution (CAS number 2052-49-5, purchased from Sigma-Aldrich, Poland) and acetonitrile (CAS number 75-05-8, purchased by Sigma-Aldrich, Poland) were used as eluents.

In differential pulse voltammetry, the synthesized chelates, sodium perchlorate solution (CAS number 7601-89-0, purchased from Sigma-Aldrich Poland) were used as the basic electrolyte, 1 mass% aqueous solution gelatin (CAS number 9000-70-8, purchased from POCH, Poland).

### 3.2. Methods

The studies were carried out to demonstrate the stability of Fe-EDDHA and Fe-EDDHSA chelates. For the analysis, in the first stage, the chromatographic method was used, which allows the content of chelates and their isomers (Fe-EDDHA) or forms (Fe-EDDHSA) to be determined. Analyses were performed by taking samples of the respective solutions daily for 30 days. The used environment corresponds to acidic and neutral soils (pH 3, 5, 7). When selecting the analysis environment, the resistance of the analytical column used (pH range 1–8) was also taken into account. In the second part of the study, the method of voltammetric analysis was used, allowing the determination of iron ions content, taking samples for analysis every day for 21 days. The analyses were performed at pH 3, 5, 7, 9, 11, 13. An aqueous medium was used in both stages.

#### 3.2.1. Chelates Synthesis

Fe-EDDHA

**Figure 15:**
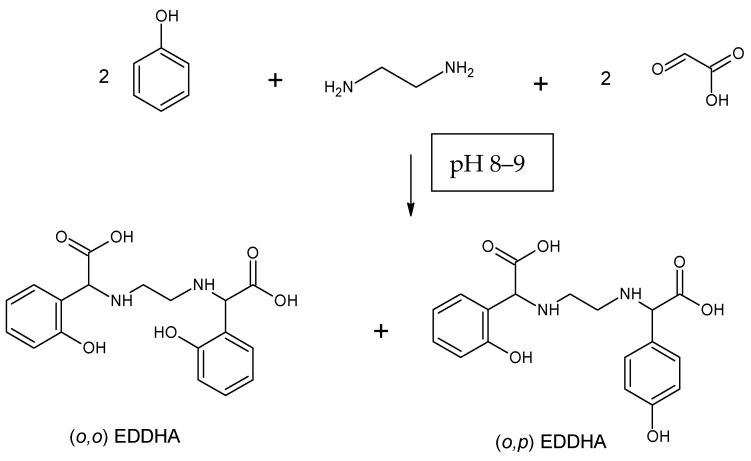
Reaction scheme for the preparation of EDDHA using the Mannich reaction.

A modified one-step Mannich reaction was used to obtain the Fe-EDDHA chelate. The synthesis was carried out under laboratory conditions. A 15-fold excess of phenol, ethylenediamine, sodium hydroxide, and 50 mass% aqueous solution of glyoxylic acid were used and the mixture was stirred for 3 h at the temperature of 70 °C. Subsequently, the mixture was cooled to room temperature and water and toluene were added. Extraction was performed twice to remove excess phenol (Figure 15) [22,28].

Iron(III) nitrate nanohydrate was added to the aqueous layer. Chelation was performed at pH 6.5. The whole was stirred for 3 h, subsequently, 50 mass% aqueous sodium hydroxide solution was added to the system so that the pH is 7–8. Under such conditions, the chelate precipitates. The resulting product was filtered off and dried.

Fe-EDDHSA

**Figure 16:**
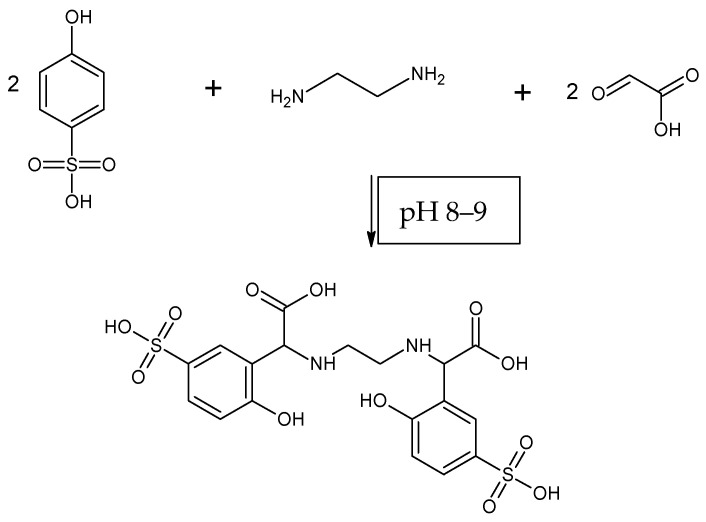
Reaction scheme for the preparation of EDDHSA using the Mannich reaction.

A one-step Mannich reaction was used to obtain Fe-EDDHSA. A slight excess of *para*-hydrosybenzosulfonic acid, ethylenediamine, glyoxylic acid, and an aqueous sodium hydroxide solution were used. The reaction was carried out for 3 h at the temperature of 70 °C (Figure 16) [28].

After cooling, iron(III) nitrate nanohydrate was added to the system. The chelation reaction was carried out for 3 h at pH 6.5. The pH was changed to 7–8 successively with an aqueous solution of sodium hydroxide. Under these conditions, Fe-EDDHSA chelate crystallizes from the solution. The solution was filtered and the resulting precipitate was dried.

#### 3.2.2. Analytical Methods

Ion Pair Chromatography

Fe-EDDHA

The Fe-EDDHA analysis was performed in accordance with EN 13368-2:2017 by ion pair chromatography. This method enables the determination of chelated iron content by EDDHA, EDDHMA, and HBED. It also makes it possible to determine the content of *d,l-rac* Fe-(*o*,*o*)EDDHA, meso isomers Fe-(*o*,*o*)EDDHA, Fe-(*o*,*p*)EDDHA, Fe-(*p*,*p*)EDDHA. The principle of the method is the formation of ion pairs. The iron chelate as an anion is added to a polar eluent (acetonitrile) containing a large cation (tetrabutyl-ammonium TBA^+^) which forms an ion pair with the chelate. The complex thus formed is bound to the non-polar stationary phase. The retention time depends on the size of the molecule and its acidity [29].

The analyses were performed using the Thermo Scientific Dionex ICS 5000+ chromatographic set (United Kingdom). A Eurospher II 100 Vertex Plus C18A 4.0 × 150 mm 5 µm column (Knauer, Deutsch) with precolumn was used. The isocratic mode was used. The eluent flow was 1 cm^3^/min. The injection volume was 20 µL. A UV–Vis detector (Dionex) was used and measurements were made at a wavelength of 280 nm. The analysis lasted 15 min. Demineralized water was used. The eluents were degassed on an Emmi 30 HC ultrasonic cleaner by EMAG AG (Germany). Each sample was filtered through NYLON syringe filters (45 µm × 25 mm).

Fe-EDDHSA

The Fe-EDDHSA analysis was performed in accordance with EN 15451 using ion pair chromatography. The ionic pair is formed with the anions of the iron chelate and the large cation (TBA^+^) contained in the polar eluent composition (acetonitrile). Such a complex is associated with a non-polar stationary phase. The elution time is related to the size of the molecule and its acidity. This method enables the separation of Fe-EDDHSA chelate and condensation products formed during the synthesis [30].

The analyses were performed using the Thermo Scientific Dionex ICS 5000+ chromatographic set (United Kingdom). A Eurospher II 100 Vertex Plus C18A 4.0 × 150 mm, 5 µm column (Knauer, Deutsch) with precolumn was used. Gradient mode was used. Two eluent solutions differing in the content of acetonitrile were used. The eluent flow was 1 cm^3^/ min. The injection volume was 20 µL. A UV–Vis detector (Dionex) was used and measurements were made at a wavelength of 480 nm. The analysis lasted 20 min. Demineralized water was used. The eluents were degassed with an Emmi 30 HC ultrasonic cleaner by EMAG AG (Germany). Each sample was filtered through NYLON syringe filters (45 µm × 25mm). The analysis of the obtained chromatograms was performed with the Chromeleon 7 software.

The content of both forms was calculated from the standard curve. The area under the peak is directly proportional to the concentration. The calculations were made in the same way as for EDDHA.

Differential Pulse Voltammetry

The content of unbound iron(III) ions was determined using differential pulse voltammetry (DPV). It is an electroanalytical technique that measures the dependence of the intensity of the current flowing through a stationary indicator electrode and the potential of this electrode. The voltammetric method is one of the most sensitive analytical methods allowing the concentration of metal ions to be determined in trace amounts. This enables the simultaneous measurement of several ions and allows the degree of metal oxidation to be distinguished [31].

The tests were performed using the AUTOLAB PGSTAT 12 apparatus with GPES software (Metrohm, Switzerland). A mercury electrode (663 VA Stand) working in SMDE mode (Static Mercury Drop Electrode) was used. The reference electrode was the silver chloride electrode and the auxiliary glass fiber electrode. The mercury drop area was approximately 0.25 mm^2^. The step potential was 0.00495 V and the modulation amplitude was 0.00255 V at 0.05 s. The equilibration time was 5 s. The deposition potential was −1.3 V at 60 s. Each measurement was repeated twice. The concentration of metal is directly proportional to the height of the current signal.

The determinations were made in the environment of the base electrolyte. The primary electrolyte was 0.1 M NaClO_4_ solution. Each analyzed sample contained solutions of iron(III) cations, chelating compound, gelatin, base electrolyte. The pH was adjusted and thus the prepared solution with a volume of 25 cm^3^ was placed in the electrolytic cells. An inert gas, nitrogen was used to remove oxygen from the system. Then a measurement was taken.

## 4. Conclusions

The paper presents the results of research related to the change in the content of unbound iron (III) ions and Fe-EDDHA and Fe-EDDHSA chelates as well as their isomers and forms. The tests were carried out in the water environment at various pH values. Fe-EDDHA and Fe-EDDHSA chelates in aqueous solution are the most stable at pH 7. Their concentration does not change over time. The content of EDDHA isomers is slightly changed. The concentration of (*ortho*, *ortho*) and (*ortho*, *para*) isomers is constant over time. The content of the isomer (*para*, *para*) decreased to about 0.1%. The amount of monomer and EDDHSA condensation products over time is constant at this pH value, respectively 48.68% and 51.32%. This chelate is stable over time and can be stored as an aqueous solution.

Fe-EDDHSA chelate was very stable in the aquatic environment over time. The content of monomer and condensation products changed during the experiment. Both the monomer and condensation products have chelating properties. Differences in the amount of both forms in the system do not affect the effectiveness of iron deficiency supplementation. At pH 7, the smallest changes in the content of unbound iron ions, chelate, monomer, and condensation products were observed. Fe-EDDHSA chelate meets all the conditions to be produced and stored in the form of an aqueous solution.

## Figures and Tables

**Table 1:** The content of unbound iron(III) ions at time *t* = 0 in the aqueous medium at different pH values in the preparation of Fe-EDDHA and Fe-EDDHSA.

pH	Fe^3+^ in Fe-EDDHA [%]	Fe^3+^ in Fe-EDDHSA [%]
3	5.32%	6.25%
5	5.86%	2.66%
7	3.30%	2.48%
9	0.39%	4.17%
11	6.01%	1.81%
13	2.56%	0.38%

**Table 2:** Content of isomers iron(III) ethylenediamine-*N*,*N*′-bis(hydroxyphenylacetic) acid in the aqueous medium at pH 3, 5, and 7 at *t* = 0.

pH	Initial Content Fe-(*o*,*p*) EDDHA [%]	Initial Content Fe-(*p*,*p)* EDDHA [%]	Initial Content *d,l-rac*-Fe-(*o*,*o)* EDDHA [%]	Initial Content *meso*-Fe-(*o*,*o)* EDDHA [%]
3	3.11	7.39	56.04	33.46
5	2.43	5.49	44.93	47.15
7	2.61	6.30	44.32	46.77

**Table 3:** Content of monomer and condensation products iron(III) ethylenediamine-*N*,*N*′-di[(2-hydroxy-5-sulfophenyl)acetic acid] in an aqueous medium at pH 3, 5 and 7 at time *t* = 0.

pH	Initial Monomer Content [%]	Initial Content of Condensation Products [%]
3	58.53	41.47
5	50.28	49.72
7	50.40	49.60

## Data Availability

Not applicable

## Sample Availability

Samples are available from the authors on request.
